# Broadening the reach of the FDA Sentinel system: A roadmap for integrating electronic health record data in a causal analysis framework

**DOI:** 10.1038/s41746-021-00542-0

**Published:** 2021-12-20

**Authors:** Rishi J. Desai, Michael E. Matheny, Kevin Johnson, Keith Marsolo, Lesley H. Curtis, Jennifer C. Nelson, Patrick J. Heagerty, Judith Maro, Jeffery Brown, Sengwee Toh, Michael Nguyen, Robert Ball, Gerald Dal Pan, Shirley V. Wang, Joshua J. Gagne, Sebastian Schneeweiss

**Affiliations:** 1 Division of Pharmacoepidemiology and Pharmacoeconomics, Department of Medicine, Brigham and Women’s Hospital and Harvard Medical School, Boston, MA USA; 2 Department of Biomedical Informatics, Vanderbilt University Medical Center, Nashville, TN USA; 3 Department of Population Health Sciences, Duke University, Durham, NC USA; 4 Biostatistics Unit, Kaiser Permanente Washington Health Research Institute, Seattle, WA USA; 5 Department of Biostatistics, University of Washington, Seattle, WA USA; 6 Department of Population Medicine, Harvard Pilgrim Health Care Institute and Harvard Medical School, Boston, MA USA; 7 Office of Surveillance and Epidemiology, Center for Drug Evaluation and Research, FDA, Silver Spring, MD USA; 8 Present Address: Johnson & Johnson, New Brunswick, NJ USA

**Keywords:** Epidemiology, Public health

## Abstract

The Sentinel System is a major component of the United States Food and Drug Administration’s (FDA) approach to active medical product safety surveillance. While Sentinel has historically relied on large quantities of health insurance claims data, leveraging longitudinal electronic health records (EHRs) that contain more detailed clinical information, as structured and unstructured features, may address some of the current gaps in capabilities. We identify key challenges when using EHR data to investigate medical product safety in a scalable and accelerated way, outline potential solutions, and describe the Sentinel Innovation Center’s initiatives to put solutions into practice by expanding and strengthening the existing system with a query-ready, large-scale data infrastructure of linked EHR and claims data. We describe our initiatives in four strategic priority areas: (1) data infrastructure, (2) feature engineering, (3) causal inference, and (4) detection analytics, with the goal of incorporating emerging data science innovations to maximize the utility of EHR data for medical product safety surveillance.

## Background

The United States Food and Drug Administration (FDA)’s Sentinel System (referred to as “Sentinel” hereafter) uses distributed analytic tools and curated real-world longitudinal health insurance claims data for more than 100 million people from participating healthcare systems to generate insights regarding the safety of medical products^1,2^. It is an important resource that informs drug labeling, drug safety communications, FDA Advisory Committee meetings, and other regulatory decisions^3,4^. Health insurance claims data currently form the backbone of Sentinel, owing to their complete capture of outpatient pharmacy dispensing records, medical encounters, and hospitalizations during well-defined periods of health plan enrollment. Reliable implementation of drug safety analyses is achieved in a timely manner within Sentinel through the Active Risk Identification and Analysis (ARIA) system, which consists of modular programs that apply sophisticated epidemiologic study designs and analyses to distributed health insurance claims data organized in a common data model^1^.

In a recent analysis of ARIA capabilities, some of the most frequently cited reasons for the inability of using the current claims-based Sentinel for safety investigations included the lack of clinical details to accurately identify health outcomes, missing or inaccurate measures of important confounding variables, or unavailability of computable phenotyping algorithms to identify study populations with acceptable accuracy^5^. Linking electronic health records (EHRs), which contain granular information related to clinical parameters, with insurance claims will likely address some of the current gaps in Sentinel’s capabilities. In 2019, Congress required the FDA to create a Medical Data Enterprise to enhance the Sentinel infrastructure by incorporating EHR data from at least 10 million lives^6^.

With all the opportunities for improvement, the inclusion of additional EHR data also introduces several methodological challenges that need to be addressed to facilitate robust analyses using a large-scale data infrastructure of combined EHR and claims data. The FDA has outlined an approach for achieving these goals in a 5-year Sentinel System strategic plan^7^. In this report, we describe a roadmap of the newly launched FDA Sentinel Innovation Center to strengthen the Sentinel System by outlining key challenges and proposed initiatives to address them.

## Identifying and incorporating fit-for-purpose data sources

**Fig. 1:**
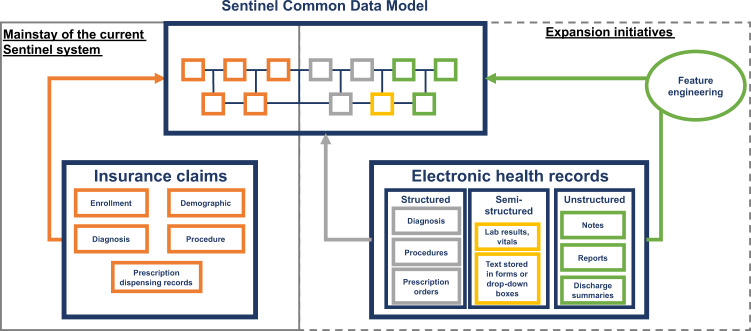
Conceptual overview of the integration of claims data and electronic health records in Sentinel. Solid box on the left indicates data elements currently available in the Sentinel common data model, dotted box on the right indicates elements from electronic health records that will be considered for inclusion.

To expand Sentinel’s access to EHR data, a first-order goal is to develop the organizational framework and establish the governance, harmonization, and quality assurance processes for ensuring high-fidelity, fit-for-purpose data to support queries of regulatory importance. To support causal conclusions, establishing a clear temporal sequence of events based on data from sources with near-complete longitudinal capture is imperative. As most EHR sources in the US lack the ability to capture data when individuals receive care outside of the contributing healthcare systems, linkage of EHRs to insurance claims, which captures longitudinal data regardless of the care settings, is necessary to understand the completeness of longitudinal data. In establishing a query-ready distributed data network containing EHRs, the Sentinel Innovation Center will address key regulatory needs including determining where to source the EHR data with standing linkage to insurance claims and defining the minimum data elements necessary in order to address use cases that are currently difficult to address. Additionally, we will outline principles and strategies for determining how to organize both the structured, semi-structured and unstructured EHR data alongside insurance claims data in a common data model to facilitate standardized query implementation (Fig. 1).

## A system rooted in a causal analysis framework

To comprehensively monitor the safety of marketed medical products, Sentinel investigations focus on both signal identification, which is intended to generate hypotheses regarding unsuspected adverse events, as well as signal refinement and evaluation, which is intended to test previously generated hypotheses and identify susceptible populations^8^. As regulatory decisions are predicated on inferring causal relations between a medical product exposure and adverse outcomes, a causal analysis framework fitting nonrandomized treatment allocation and secondary data is needed^9^. Even for signal identification based on data mining methods, attention to principles including clearly established temporality of confounder assessment preceding exposure followed by outcome surveillance, paired with analytic strategies that reduce confounding, is required to limit the number of spurious signals^10–12^. For signal refinement and evaluation, in addition to these important principles, further considerations include explicitly specifying comparison groups, study populations, and specific outcome definitions ideally contemplated in a hypothetical ‘target’ trial which investigators then emulate in the secondary data of Sentinel^13,14^. Analyses further should be accompanied by robustness evaluations to address the consistency of evidence with respect to alternative investigator decisions in study design, analysis, or variable measurement. To clarify challenges in developing and applying causal methods that leverage both claims and EHR data sources, the Sentinel Innovation Center will develop a causal analysis framework proposing a stepwise process that systematically considers key choices with respect to design and analysis that influence the validity of studies conducted with nonrandomized data. A standardized “industrial” process that will be outlined in this framework will serve as a valuable tool to inform the conduct and assessment of the quality of nonrandomized studies of drug-outcome evaluation.

## EHR data-specific innovation needs

**Fig. 2:**
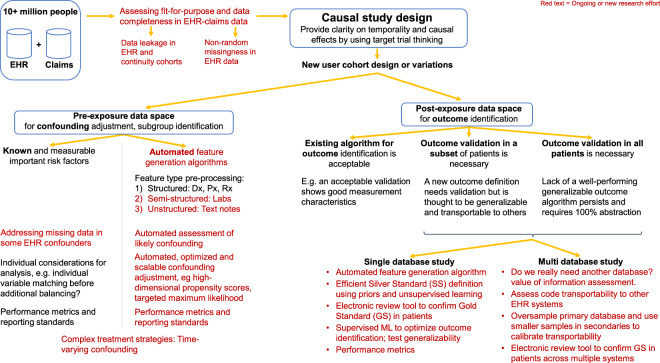
Methodological research needs to support FDA safety decision-making with linked EHR-claims data. A summary of key infrastructure, design, and measurement challenges is described. Red text indicates ongoing or future research activities.

**Fig. 3:**
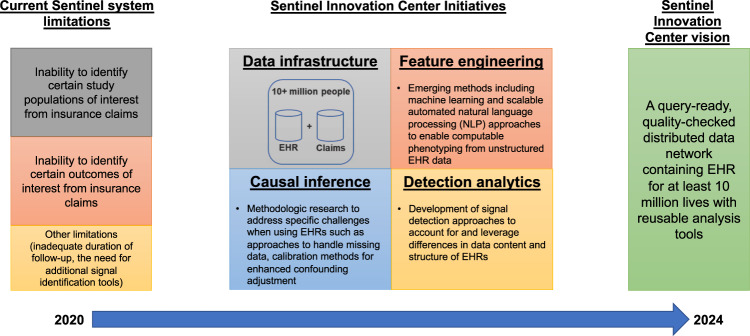
The Sentinel Innovation Center initiatives and vision. Arrow at the bottom indicates timeline for the proposed activities.

While EHR data offer great promise for improving Sentinel’s capabilities, including improvements in computable phenotyping and confounding control, they also bring a range of measurement challenges that must be addressed. Expanded measurement procedures and analytic approaches are outlined in Fig. 2, identifying a range of Innovation Center projects. Briefly, we focus on distinct measurement challenges (1) to determine patient characteristics in the pre-exposure space (left side) where automated feature identification processes may feed directly into data-adaptive confounding adjustment procedures^15^ and (2) to identify and expeditiously validate health outcomes of interest in the postexposure space (right side) by augmenting claims-based algorithms with EHR-based data that may be structured, semi-structured, or unstructured. The Innovation Center has formed four main domains as strategic priority initiatives to deliver a query-ready, large-scale data infrastructure of combined EHR and claims data: data infrastructure, feature engineering, causal inference, detection analytics (Fig. 3).

## Data infrastructure development

EHR data are heterogeneous in their content, structure, completeness, and quality. To enable efficient and timely querying of EHRs to support surveillance activities, pre-processing to make them query-ready is critical. To achieve this, the Sentinel Innovation Center is developing a principled approach to extend the Sentinel Common Data Model to include new data elements from structured and unstructured EHR data (Fig. 1). While common data models can help to impose a standard organization of the data across multiple sites, mapping of source values into the model can lead to omissions, data errors, and other data consistency issues^16^. Even though a data model standardizes the name of data elements, a lack of semantic interoperability across sites may remain. To address this challenge, we will investigate approaches to detect and mitigate data consistency issues and to develop and harmonize data across multiple EHR data sites. Further, we will focus on the development of a set of data quality metrics and approaches for the integration of structured and unstructured data elements from EHR into a common data model to facilitate reliable analyses of medical product outcomes using these data.

## Feature engineering approaches

Health insurance claims data, which have been the primary source of data within the Sentinel System, are highly structured with all information stored using standard terminologies such as International Classification of Diseases (ICD) codes for diagnoses and National Drug Codes (NDC) for filled prescriptions. Some of the EHR-based data such as administrative data, medication orders, and most laboratory testing results are recorded as structured or semi-structured fields, but a vast amount of potentially useful information is stored in visit notes (e.g., narrative descriptions of a patient’s signs and symptoms, family history, social history), radiology reports or images, and discharge summaries as unstructured data. Substantial engineering is needed to identify features from unstructured data that can be extracted and organized as structured data.

Natural language processing (NLP) and automated feature extraction are essential mechanisms to support scalable computable phenotyping from EHRs in signal detection and signal refinement activities. Development of automated feature extraction workflows that allow for time-contextualization is critical to enable the determination of temporality in confounder, exposure, and outcome assessment in Sentinel queries. However, there are numerous challenges in using these approaches at scale in national consortia. NLP tool performance between sites has been a challenge due to systematic data changes. Tool development further needs to be tailored to the eventual application of the derived features. For instance, identification of health outcomes of interest needs focused tools to ensure optimal performance characteristics including positive predicted value and specificity, while identification of potential confounders may utilize more flexible tools utilizing unsupervised approaches for high-dimensional feature vector generation. To address these challenges, the Sentinel Innovation Center has initiated a range of activities. The first set of projects aims to develop and validate algorithms for the identification of health outcomes of interest using focused NLP tools combined with machine learning approaches to identify complex concepts such as suicidal ideation. This work will expand on prior Sentinel activities that have demonstrated the proof of concept for use of algorithmic approaches in improving outcome identification^17–19^ and focus on developing a general framework for efficient and standardized processes. Another set of activities aim to build a semi-automated system of confounding adjustment that uses generalized NLP tools to enable large-scale extraction of features that appear sequentially and may serve as indicators of patients’ health trajectory. Finally, initiatives are also underway to improve the generalizability and transportability of NLP approaches across sites using statistical learning approaches^20^.

## Causal inference methodology

Typical Sentinel investigations for drug safety conducted using insurance claims data sources have relied on restrictive study designs such as the active comparison of new user designs to achieve internal validity given the availability of large underlying populations; however, the use of EHR-claims linked sources may require alternate design choices, such as prevalent new user design^21^ to accommodate relatively smaller underlying populations. Tradeoffs when deviating from traditional design choices to accommodate available data assets need to be thoroughly investigated. Other unique challenges when using EHR for the outcome and confounder measurement include nonrandom missingness, or similarly the selective presence of data such as medical tests that may be ordered in light of the patients’ prognosis, or incompleteness that occurs when outcomes recorded outside of the care systems are not available (“data leakage”). The Sentinel Innovation Center will focus on characterizing these challenges and developing strategies, methods, and tools to address them.

Residual confounding due to selection into treatment groups driven by outcome risk factors is another salient challenge in nonrandomized studies. The issue is accentuated when using data that lack clinical granularity such as insurance claims. Availability of EHR sources that contain richer clinical information on factors not readily available in claims hold promise to improve confounding adjustment in nonrandomized studies. The Sentinel Innovation Center will evaluate the feasibility of improved confounding adjustment from EHR-based variables through a combination of automated feature generation algorithms and advanced statistical and machine learning approaches such as Super Learner and Targeted Maximum Likelihood Estimation (TMLE)^22,23^. Super Learner, which is an ensemble algorithm for predictive modeling, can data-adaptively model confounder summary scores, such as the propensity score, and the outcome to address model misspecification in the setting of complex and high-dimensional data settings of EHRs. TMLE can incorporate data-driven methods for high-dimensional confounder selection to empirically identify confounder information not specified by investigators. Developing scalable tools to implement these innovative methods in real-time will enhance the ability of Sentinel to address the common threat of confounding. Additionally, the Innovation Center will also investigate methods such as negative control outcomes or exposures and enhance existing tools for quantitative bias analyses^24^ to better understand the robustness of findings, including the impact of residual confounding.

Availability of additional clinical information in EHR further opens new opportunities to (1) identify outcomes that are generally not identifiable with claims data alone or (2) to efficiently validate claims-based algorithms in a subset of patients with EHR data available. The use of advanced methods based on machine learning and NLP to expedite outcome identification or validation have the potential to increase the efficiency of traditional drug safety evaluations. For instance, data-adaptive validation techniques where human experts review batches of patient charts for endpoint validation based on claims or EHR data could be iteratively used to train algorithms to inform the selection of cases that have a higher likelihood of being a true positive in the next batch^25^. Incorporating NLP-assisted technology that can sift through patient charts and present the most relevant chart based on pre-specified key terms to human expert reviewers can add further efficiency to the process. The Sentinel Innovation Center will consider methodologic research to improve outcome identification and validation, which is a vital requirement for reliable evaluation of medication safety in Sentinel.

## Detection analytics

Data mining approaches such as TreeScan have been developed in insurance claims data for signal detection in the Sentinel infrastructure based on the grouping of ICD diagnosis codes into hierarchical levels^10^. EHRs offer a potentially promising complementary source of information for medication safety signal detection but may require tailored approaches to account for and leverage differences in data content and structure compared to insurance claims. Specifically, unstructured clinical narratives recorded in EHR may provide more complete capture of subtle adverse events that may not trigger formal coding or medical interventions, aspects that are observable in claims data. The addition of detailed information presents an opportunity to expand signal detection efforts that are currently used in Sentinel. While NLP-based identification of adverse events from unstructured clinical notes is feasible with currently available methods, relational identification of newly occurring adverse events in a temporal sequence to specific medication exposures is complex and the subject of active research^26^. The Sentinel Innovation Center plans to develop a methodological framework and conduct empirical evaluations to identify and test the most promising approaches for EHR-based signal detection.

## Demonstration projects to calibrate the validity of RWE studies

The Sentinel Innovation Center will launch several demonstration projects focusing on use cases typical for Sentinel; associations of medications for chronic conditions with infrequently observed outcomes where EHR data linkage has the potential to offer enhancements with respect to population identification, confounding adjustment, or outcome measurement. In these demonstration projects, we will emulate the corresponding target trial using principles and methodology developed across several Sentinel Innovation Center initiatives described above. When available, we will benchmark results against those observed in the corresponding pivotal clinical trial findings^14^.

## Transparency and reproducibility of process and implementation

Transparent communication of study specifications in a pre-registered protocol, akin to a prospective trial protocol, is a feature that helps increase confidence in results of hypothesis-confirming studies from nonrandomized studies using secondary EHR-claims data. A focus of the Sentinel Innovation Center will be to facilitate EHR adaptation of existing tools such as the structured template and reporting tool for real-world evidence (STaRT-RWE) planning, implementation and communication template^27^, to prepare and be able to register study protocols for future Sentinel queries. Additionally, adherence to principles for transparent reporting of analytic assumptions and diagnostics in line with good reporting practices^28^ as well as transparent research practices including sharing of the analytic codes will be emphasized.

## Vision for strengthening the current system through fostering innovation

Enhancements in the current Sentinel system proposed by the Sentinel Innovation Center that are summarized in this report will be achieved through strategic investment in focused research projects working in harmony. The new system containing granular clinical information through the integration of EHRs will broaden the scope of the current system by expanding on the types of medication safety and effectiveness questions that are insufficiently addressed by insurance claims only. This may include questions where insurance claims data do not have sufficient information to identify the underlying population of interest, the outcome of interest, or a critical confounder to support a valid comparative analysis. Additionally, this system will also strengthen investigations that are possible to implement with insurance claims only through activities such as enabling extensive clinical characterization in a subset of patients selected for treatment with different medications and facilitating outcome algorithm development and validation for implementation in broader insurance claims-based network.

## Conclusion

The FDA Sentinel Innovation Center has outlined a set of initiatives and a project portfolio^29^ to deliver a query-ready distributed data network containing EHR linked to claims and reusable analysis tools to enhance the capabilities of the current system. These initiatives will incorporate data science innovations, such as scalable natural language processing and machine learning, to maximize the potential of EHR for medical product safety surveillance.
